# Transformational leadership and flourishing in Portuguese professional firefighters: The moderating role of the frequency of intervention in rural fires

**DOI:** 10.3389/fpsyg.2023.1076411

**Published:** 2023-02-13

**Authors:** André Maio, Maria José Chambel, Laura Carmona

**Affiliations:** ^1^Centro de Psicologia Aplicada do Exército, Exército Português, Queluz, Portugal; ^2^CicPsi, Faculdade de Psicologia, Universidade de Lisboa, Lisbon, Portugal

**Keywords:** transformational leadership, flourishing, firefighters, rural fires, well-being

## Abstract

**Introduction:**

The main objective of this study is to analyze the effect of transformational leadership on firefighters’ well-being and understand the moderating role of the frequency of intervention in rural fires in this relationship.

**Methods:**

A total of 90 responses of Portuguese professional firefighters were analyzed in two waves (T1 and T2) separated by a period of 3 weeks during which the frequency of intervention in rural fires were also recorded on a daily basis.

**Results:**

There is a direct and positive effect, albeit small, of the transformational leadership dimensions on flourishing. Moreover, the frequency of intervention in rural fires amplified the effect of individual consideration on this wellbeing indicator, and it was observed that the more frequent the firefighters intervene in rural fires, the stronger the effect of this leadership dimension on their flourishing.

**Discussion:**

These results contribute to the literature to the extent that they highlight the role of transformational leadership in promoting well-being in high-risk professions, thus supporting the assumptions of the Conservation of Resources Theory (COR). Some practical implications are presented, as well as limitations and suggestions for future studies.

## Introduction

The promotion of occupational health and well-being has motivated a cross-cutting interest in the work context (Danna and Griffin, 1999). One of the main goals of organizations is to have happy employees, with high levels of well-being and high-performance rates (Nielsen et al., 2017). However, there is still much to be achieved in this regard. According to recent statistical data, in the United Kingdom, the number of employees reporting problems related to stress, depression, and anxiety has increased in recent years (e.g., in 2019/20 they accounted for 51% of all work-related health problems; Health and Safety Executive, 2020). These health problems are more prevalent in the civil service (e.g., firefighters), where higher levels of stress compared to employees in other employment areas (Health and Safety Executive, 2020) are observed. Furthermore, it has also been reported that the main triggers of these health problems are related to: high workload; tight task completion deadlines; high levels of responsibility; and lack of supervisory support (Health and Safety Executive, 2020). Thus, the supervisory support (such as well-being-promoting leadership behaviors; Montano et al., 2017; Inceoglu et al., 2018) and the professional context (which in certain work environments, as is the case with emergency professionals, such as firefighters, is unstable and highly demanding; Adler and Castro, 2013; Ângelo and Chambel, 2015) have a clearly important impact on well-being and on occupational health. Therefore, considering the professional environment of firefighters, the relationship between well-being promoting leadership and the specific context of this population calls for further examination.

In fact, transformational leadership may be an important vehicle to achieve well-being at work (e.g., Sivanathan et al., 2004), even in highly demanding contexts (Bass, 1998). Transformational leadership may be understood as the process by which the leader makes the subordinates go beyond their immediate self-interests (Bass, 1999; Bass and Riggio, 2006), by means of: *idealized influence*—the leader is regarded as a role model with high moral and ethical standards, and in emergency situations subordinates have to trust that the leader is capable of making the best possible decision in difficult situations; *motivational inspiration*—the leader provides meaning, challenging subordinates to excel and in the context of firefighting, for example, will be crucial that after stressful and/or disturbing incidents leader promotes the debriefing and subordinates remain confident in relation to future occurrences, with an attitude of overcoming adversity; *intellectual stimulation*—the leader promotes initiative and creativity, involving subordinates in problem-solving and in the context of firefighting leaders need promote the development of subordinates’ skills, so that, in emergency situations, they can take advantage of the strengths of each team member; and *individualized consideration*—the leader takes the needs of each individual into consideration, acting as a mentor, accepting individual differences, and personalizing interactions with his/her subordinates and in the context of firefighting the leader has to support subordinates often exposed to adversity, and be available to listen them and act in accordance with their requests or complaints.

Although different studies have proven the advantageous effect of transformational leadership on subordinate’s well-being and ill-being (stress and burnout), there is a scarcity of studies with emergency professionals and with firefighters. In both the systematic review of Arnold (2017) and the systematic review of Harms et al. (2017), only one study with professionals of emergency (i.e., police officers) showed that transformational leadership (i.e., inspirational motivation dimension) have a negative effect on the subordinate ill-being (i.e., emotional exhaustion, a dimension of burnout). Thus, it is not known whether transformational leadership is, in fact, effective in ensuring the well-being of emergency professionals, namely firefighters. This knowledge is especially important because firefighters are exposed to high operational demands (e.g., demands during the intervention in fire operations), that may lead to a decline in their well-being (Airila, 2015).

Therefore, this two-wave study, conducted with Portuguese professional firefighters, aims, firstly, to analyze the effect of the different dimensions of transformational leadership on subordinates’ well-being (i.e., flourishing). In addition, it seeks to ascertain the extent to which the afore-mentioned relationship is affected by the high operational demands to which these professionals are subject during periods of rural firefighting.

The current study has three innovations. First, it is a contextualized analysis to clarify to what extent transformational leadership dimensions can be used to promote well-being in emergency professionals, namely firefighters. Second, considering that these professionals face many demands in their daily lives, we analyzed to what extent this effect of transformational leadership on subordinate’s well-being was especially relevant in situations of high demands. In this way, we analyzed the moderating effect of rural fires frequency in the aforementioned relationship. Third, it uses a longitudinal design, whereas most of the previous studies is cross-sectional (Arnold, 2017; Harms et al., 2017). In fact, only longitudinal studies allow an understanding of the evolution of the relationship between the variables, thus, controlling for constant individual heterogeneity over time.

## Theoretical framework

### Transformational leadership and flourishing

Conservation of Resources Theory (COR, Hobfoll, 2002) describes how people try to obtain, retain, and protect resources (i.e., the objects, personal characteristics, and physical, psychological, and social conditions that individuals value because they allow them to achieve their goals), and stress occurs when individuals have the risk of losing, or actually lose, such resources, since, when individuals perceive the presence of a stressor, they are led to invest resources in an attempt to cope with these stressful situations (Hobfoll, 2002). However, a way to combat this negative effect on well-being is to make resources available. In fact, according to the assumptions of the COR, those who have more resources are not only less vulnerable to the effect of stressors, given their greater ability to cope with the demands and challenges of their professional activity, but they are also better equipped to mobilize new resources and experience their professional activity as a positive occurrence that provides pleasure, satisfaction, and enthusiasm.

In professions such as firefighting, where demands cannot be removed and may even be increased in extreme situations (Geier, 2016), it is essential to provide these professionals with resources that mitigate the negative effects of high operational demands (such as the participation in rural fires), ensuring a reduction of burnout and an increment of well-being (Bakker and Demerouti, 2007). In fact, not only are firefighters in a better position to successfully manage their demands when they are equipped with high levels of resources (Tuckey et al., 2012), but also the consequences of work-related critical incidents in firefighters’ well-being are determined by the availability of resources. These resources can be provided in different manners, e.g., through supervisory support, organizational support, constant feedback, clear objectives, autonomy (Hobfoll et al., 2018), professional development opportunities, and participation in decision-making processes (Halbesleben et al., 2014). Supervisory action (i.e., leadership style) is a crucial element for the promotion of such resources in these professionals (Hobfoll et al., 2018).

The leader’s actions are based on the distribution of valuable resources so that the subordinates can perform their professional activity. On the one hand, they prevent the loss or threat of the loss of resources, which characterize high demand situations, thus preventing the onset of strain such as burnout (Harms et al., 2017). By guiding the subordinates to pursue new avenues in problem-solving and providing a positive alternative view of the scenarios with which they are confronted, their experience of stress can be reduced (Bass and Bass, 2008; Diebig et al., 2016). For example, leader might promote periodic discussion about different constraints and obstacles that could occur in a rural fire that promote the perception of firefighters’ efficacy that equip them to face the demands of this situation. In fact, empirical results demonstrated that emergency professionals who reported supervisor support (Bacharach et al., 2008) tended to show less stress. On the other hand, the acquisition of valuable resources provided by a leader’s actions fosters the acquisition of more resources. These resources are useful to achieve a sense of achievement, satisfaction, and resilience in the face of difficulties (Chen et al., 2018). The transformational leader focuses on the development of subordinates (Bass, 1990; Nanjundeswaraswamy and Swamy, 2014), seeking to meet their intrinsic needs (Judge and Piccolo, 2004), inspiring and helping them develop their value system to grow as people (Ismail et al., 2009), encouraging them to look at problems in light of new perspectives and offering them support during their problem-solving process (Bass and Avolio, 1990), and motivating them through charisma, support, and the implementation of new processes that are more rewarding and productive to constantly develop and improve their performance, which not only leads to better results, greater satisfaction (McClanahan, 2020) and better mental health (Kuoppala et al., 2008; Kaluza et al., 2020), but also provides for the well-being of his/her subordinates (Arnold, 2017).

Several theoretical conceptions (De Simone, 2014; Fisher, 2014) have focused on describing the dimensions of employees’ well-being (Dodge et al., 2012), following two distinct trends. On one hand, the idea of subjective well-being, which involves an affective (happiness) and a cognitive (life satisfaction) side (Diener et al., 1998); on the other, an idea that is more connected to the component of psychological functioning and human development, which emphasizes autonomy, self-acceptance, and personal growth (Ryff, 2014). Thus, it is argued that well-being is a multidimensional concept that encompasses the emotional, psychological, and social component, and can be defined as the “balance point between an individual’s pool of resources and the challenges faced” (Dodge et al., 2012, p. 230). Diener et al. (2009) suggest a component that ties together these different trends, referred to as psychological well-being, in an attempt to reflect the ideal human functioning. According to these authors, there are universal psychological needs (e.g., competence, relationships, and self-acceptance), social and psychological capital needs (e.g., interest, purpose, and meaning), and needs to help others and needs to engage in meaningful activities.

Based on this concept of psychological well-being, the empirically updated contribution of Diener et al. (2010) suggests a new indicator of well-being, namely psychosocial flourishing. This variable is reflected in a positive level of psychological functioning through personal growth, and encompasses adequate functioning (e.g., “doing” what one likes) and feeling good (e.g., “being” happy; Diener et al., 2010). Flourishing reflects self-perceived success in terms of relationships, self-esteem, purpose, and optimism (Diener et al., 2010). It may be related to personal development and fulfillment and simultaneously contribute in social terms to the satisfaction of others (Marks and Shah, 2004). Thus, a likely connection may be established between transformational leadership, regarded as a source that increased subordinates’ resources (Hobfoll et al., 2018) that contribute to their ability to cope with professional demands, which is essential for their adaptation to change, development, and experience of well-being (Dodge et al., 2012; Arnold, 2017). Therefore, transformational leadership may be an important vehicle through which firefighters may achieve flourishing. Hence, the following research hypothesis was established:

*Hypothesis 1*: Transformational leadership positively influences flourishing.

### The frequency of intervention in rural fires as moderator

The environment in which firefighters intervene is dangerous and potentially exposed to traumatic events (Cowlishaw et al., 2020), and they are subject to high demands resulting from their mission (Ângelo, 2010). The specific case of rural firefighting requires that these professionals have an enhanced capacity for readiness, sacrifice, and adaptation to extreme conditions (Campbell et al., 2010), which strongly affects their health (e.g., Psarros et al., 2018; Cowlishaw et al., 2020). This extreme context stresses the importance of leadership behaviors on the part of supervisors (Hannah et al., 2009; Campbell et al., 2010; Geier, 2016) who, in addition to their coordinating and managerial tasks based on objective and clear guidelines, also need to focus on promoting the quality of life and protecting the well-being of their subordinates (Ângelo, 2010).

According to the COR, demands make resources more important (Hobfoll et al., 2018). In fact, in situations characterized by a high workload and high pressure (such as rural fires), individuals exert more effort thus requiring a high investment of their resources (Kaluza et al., 2020). However, when individuals do not have the necessary resources to cope with these demands, their well-being is negatively affected as there is no possibility of replacing the resources invested (Hobfoll et al., 2018). On the other hand, in these situations of loss or threat of loss of resources, environmental and social conditions (e.g., leadership) play an important role in obtaining resources as they stimulate resilience in individuals, thus enhancing their ability to obtain more resources (Hobfoll et al., 2018). Therefore, a resource gain is more noticeable in a context of resource loss and leadership, as a process providing subordinates with the resources required to perform their professional activity, will play a more prominent role in more demanding situations (such as rural fires; Ângelo and Chambel, 2015).

In fact, some studies have supported the assumption that the transformational leader in a context of high demands is able to block losses and promote subordinates’ acquisition of resources. Hentrich et al. (2017) showed how transformational leadership amplified personal resources, mitigated more severe work demands, and promoted subordinates’ well-being. In a review on leadership in extreme contexts, Hannah et al. (2009) highlighted that when shifting from a less rigorous to a more demanding context, leadership needs tend to differ and are particularly necessary when subordinates feel they are lacking the resources to cope with the situation. Furthermore, this moderating effect of the frequency of intervention in rural fires, in different contexts, may also be inferred from the work of Campbell et al. (2010) who state that trust in the leader is more relevant in extreme contexts. In sum, depending on the context, the relationship between supervisory leadership and firefighters’ well-being may be affected by the change and scale of the frequency of intervention in rural fires. Thus, it is expected that:

*Hypothesis 2*: The frequency of intervention in rural fires will moderate the positive influence between transformational leadership and flourishing, to the extent that the influence of transformational leadership on flourishing will be stronger in high demand over lower demand situations.

## Methods

### Procedure and sample

The data for this study was collected in Portugal following the establishment of contact with each fire station by email, in which the study’s aims were presented and the participation of the professional firefighters that worked in intervention team’s expert in rural fires requested. A videoconference was held to present the research procedure and objectives to the firefighters at the stations interested in participating in the study, and the informed consent was distributed and signed by those wishing to be included. They were also able to ask questions and the researcher provided clarification. The data were collected through the participants’ responses to online questionnaires using the SurveyMonkey platform.

In July 2020, the participants responded to a questionnaire in which they assessed their team manager’s transformational leadership and their own flourishing, and 3 weeks later they responded to an identical questionnaire. Between these two waves, the participants took note of the frequency of their participation in rural fires on a daily basis. Participation in the study was voluntary and the participants’ anonymity was guaranteed (e.g., instructions were given to create an identification code exclusively known to the participants themselves), through which it was possible to establish a correspondence between the responses of the same participant.

We obtained a convenience sample consisted of 90 participants, all of whom were professional firefighters in intervention team’s expert in rural fires with a stable direct manager. The sample was mostly composed of male firefighters (86.7%). In terms of age, there was a 30 year range from 22 to 52 years and a significant percentage of the firefighters were aged between 37 and 40 years (36.7%), and only 11 (1%) were above 45 years. Most of the participants (62.2%) were married or cohabiting while 37.8% were single or separated. All the respondents had at least 2 years of professional experience as a firefighter.

### Measures

#### Transformational leadership

The Portuguese version of the *Multifactor Leadership Questionnaire* (MLQ; Avolio et al., 1996), which had already been used in a previous study (e.g., Salanova et al., 2011), was used to measure this variable. This Portuguese version used four items in each four dimensions of transformational leadership and the subordinates appraise the leadership behavior: idealized influence attributed (α_T1_ = 0.76 and α_T2_ = 0.92; e.g., *My leader acts in ways that build others’ respect for me*); inspirational motivation (α_T1_ = 0.87 and α_T2_ = 0.94; e.g., *My leader expresses confidence that goals will be achieved*); intellectual stimulation (α_T1_ = 0.76 and α_T2_ = 0.92; e.g., *My leader gets others to look at problems from many different angles*); individualized consideration (α_T1_ = 0.74 and α_T2_ = 0.87; e.g., *My leader considers each individual as having different needs, abilities and aspirations from others*). The participants were asked to assess the frequency with which the behaviors expressed by the items were displayed by their direct supervisor, using a five-point Likert scale (*1 = Never, 2 = Rarely, 3 = Sometimes, 4 = Very often*, and *5 = Always*). Higher scores corresponded to high levels of transformational leadership behaviors.

#### Flourishing

To assess this indicator of well-being, the validated version of the *Flourishing Scale* (Diener et al., 2010) for the Portuguese population (Silva and Caetano, 2013) was used. Examples of items are: *I lead a purposeful and meaningful life*, and *I am competent and capable in the activities that are important to me*. The participants were asked to assess the extent to which they agreed with the statements of each item on a five-point Likert scale (*1 = I strongly agree, 2 = I agree, 3 = I neither agree nor disagree, 4 = I disagree*, and *5 = I strongly disagree*). In the original version, lower scores correspond to a greater perception of well-being however to ease interpretation, presenting all the measures in a positive direction (i.e., the higher the score the higher the level of flourishing), the items were reversed. The 8 items that measured this variable displayed good internal consistency in both waves (α_T1_ = 0.86 and α_T2_ = 0.93).

#### The frequency of intervention in rural fires

To measure this variable, participants were asked on a daily basis and for a consecutive period of 15 days to indicate the *number of rural fires in which you participated*. The total number of rural fires in which each participant had been involved during this period was calculated.

#### Control variables

According to a recent review by Bernerth et al. (2018), in the specific case of the study of leadership, most control variables (e.g., demographic—gender, age) relate weakly to the overall leadership constructs studied (e.g., transformational leadership). It is suggested that most of these variables should not be included as control variables since they lack relevant theoretical and/or empirical support.

## Results

### Mean values, standard-deviation and correlations between the variables under study

Table 1 shows the mean (M) and standard deviation (SD) of the variables studied. In general terms, the firefighters appear to estimate their team manager’s transformational leadership behaviors, in all the dimensions assessed, as being frequent in both waves. A significant decrease in transformational leadership behaviors at T2 is also observed compared to T1 (idealized influence attributed *t* = 2.8, *p* < 0.01; motivational inspiration *t* = 3.0, *p* < 0.01; intellectual stimulation *t* = 3.5, *p* < 0.01; and individualized consideration *t* = 2.7, *p* < 0.01). Regarding flourishing, the firefighters appear to display high levels of well-being in both waves. In this variable, there is a slight decrease in the firefighters’ level of perception of well-being from T1 to T2, however, this difference is not significant (*t* = 0.88, *p* = 0.38). Regarding the variable related to the frequency of intervention in rural fires, these firefighters were engaged in fighting an average of 11.49 (SD = 7.88) rural fires.

**Table 1:** Mean, SD, and Correlations (*r*) between the variables under study.

	Mean	SD	*r*									
			1.	2.	3.	4.	5.	6.	7.	8.	9.	10.
1. Idealized influence attributed _T1	3.56	0.83										
2. Motivational inspiration _T1	3.61	0.84	0.79^**^									
3. Intellectual stimulation _T1	3.54	0.74	0.76^**^	0.77^**^								
4. Individualized consideration _T1	3.48	0.83	0.83^**^	0.77^**^	0.85^**^							
5. Flourishing_T1	4.19	0.44	0.09	0.15	0.13	0.13						
6. Idealized influence attributed _T2	3.33	0.88	0.62^**^	0.57^**^	0.50^**^	0.56^**^	−0.10					
7. Motivational inspiration _T2	3.36	0.94	0.63^**^	0.62^**^	0.52^**^	0.58^**^	−0.06	0.93^**^				
8. Intellectual stimulation _T2	3.27	0.83	0.58^**^	0.62^**^	0.56^**^	0.55^**^	−0.09	0.91^**^	0.85^**^			
9. Individualized consideration _T2	3.27	0.84	0.57^**^	0.49^**^	0.54^**^	0.59^**^	−0.04	0.88^**^	0.87^**^	0.90^**^		
10. Flourishing_T2	4.14	0.52	0.23^*^	0.26^*^	0.24^*^	0.23^*^	0.39^**^	0.19	0.18	0.11	0.16	
11. Rural Fires	11.49	7.88	−0.08	−0.03	0.02	−0.01	−0.09	−0.11	−0.12	−0.00	−0.06	−0.28^**^

*N* = 90. *ρ < 0.05 (two-tailed); **ρ < 0.001 (two-tailed).

According to the correlation matrix (Table 1) it may be observed that all the variables related to transformational leadership (i.e., idealized influence attributed, motivational inspiration, intellectual stimulation, and individualized consideration) at T1 are significantly and positively related to flourishing at T2, indicating that when there are more transformational leadership behaviors exhibited by supervisors at T1 there are also higher levels of flourishing in subordinate firefighters at T2. However, the results showed that the transformational leadership dimensions at T1 are not significantly correlate with flourishing at T1. Concern the variable related to participation in rural firefighting, a significant relationship can be noted between the frequency of intervention in rural fires and flourishing at T2 (*r* = −0.28, *ρ* < 0.001).

### Hypothesis testing

Hypothesis testing was performed, applying model 1 of the computational tool—*Process* (Hayes, 2018) for SPSS, which analyzes models with moderation effect. In all models the level of dependent variable (flourishing) on T1 was controlled.

Hypothesis 1 of the present study assumed a direct and positive effect of transformational leadership on flourishing. In the case of the *idealized influence attributed* dimension, Table 2 shows that it is not supported, as the relationship is not significant with the flourishing dependent variable (*β* = 0.11, *p* > 0.05). But even so it is still very close to the 95% confidence interval (*β* = 0.11, *p* = 0.07).

**Table 2:** Analysis of the effect and moderation between the variables under study—Idealized influence attributed dimension.

	*Flourishing*_T2 (*R*^2^ = 0.26; *p* < 0.001)			
	*β*	SE	*t*	*p*
Idealized influence attributed 1	0.11	0.06	1.83	0.07
Flourishing_T1	**0.42**	**0.11**	**3.76**	**< 0.001**
Rural Fires (RF)	**0.02**	**0.01**	**2.44**	**< 0.05**
Idealized influence attributed 1*RF	0.01	0.01	1.02	0.31

*N* = 90. Idealized influence attributed 1 = Idealized Influence Attributed at T1.

β, unstandardized regression coefficient. The bold values provided in table are intended to signal significant relationships.

According to Table 3, which analyzes the *motivational inspiration* dimension, it may be observed that Hypothesis 1 is supported since the relationship is significant with the flourishing dependent variable (*β* = 0.12, *p* < 0.05). Only in one of the analyses are the values not included in the 95% confidence interval, but they coincide with this same limit (*β* = 0.12, *p* = 0.05).

**Table 3:** Analysis of the Effect and Moderation between the Variables under Study – Motivational Inspiration dimension.

	*Flourishing*_T2 (*R*^2^ = 0.26; *p* < 0.001)			
	*β*	SE	*t*	*p*
Motivational Inspiration 1	**0.12**	**0.06**	**2.27**	**< 0.05**
Flourishing_T1	**0.41**	**0.11**	**3.64**	**< 0.001**
Rural Fires (RF)	**−0.02**	**0.01**	**−2.60**	**< 0.05**
Motivational Inspiration 1*RF	0.01	0.01	0.92	0.35

*N* = 90. Motivational Inspiration 1 = Motivational Inspiration at T1.

β, unstandardized regression coefficient. The bold values provided in table are intended to signal significant relationships.

Considering Table 4, Hypothesis 1 is also observed to be significantly supported in the relationship between the *intellectual stimulation* dimension and the flourishing dependent variable (*β* = 0.15, *p* < 0.05).

**Table 4:** Analysis of the effect and moderation between the variables under study—intellectual stimulation dimension.

	*Flourishing*_T2 (*R*^2^ = 0.26; *p* < 0.001)			
	*β*	SE	*t*	*p*
Intellectual Stimulation 1	**0.15**	**0.07**	**2.21**	**< 0.05**
Flourishing_T1	**0.40**	**0.11**	**3.64**	**< 0.001**
Rural Fires (RF)	**−0.02**	**0.01**	**−2.80**	**< 0.05**
Intellectual Stimulation 1*RF	0.01	0.01	1.27	0.21

*N* = 90. Intellectual Stimulation 1 = Intellectual Stimulation at T1.

β, unstandardized regression coefficient. The bold values provided in table are intended to signal significant relationships.

Finally, based on Table 5, Hypothesis 1 is also significantly supported in the relationship between the *individualized consideration* dimension and the flourishing variable (*β* = 0.14, *p* < 0.05).

**Table 5:** Analysis of the effect and moderation between the variables under study—individual consideration dimension.

	*Flourishing*_T2 (*R*^2^ = 0.26; *p* < 0.001)			
	*β*	SE	*t*	*p*
Individual Consideration 1	**0.14**	**0.06**	**2.40**	**< 0.05**
Flourishing_T1	**0.41**	**0.11**	**3.76**	**< 0.001**
Rural Fires (RF)	**−0.02**	**0.01**	**−2.67**	**< 0.05**
Individual Consideration 1*RF	**0.02**	**0.01**	**2.13**	**< 0.05**

*N* = 90. Individual Consideration 1 = Individual Consideration at T1.

β, unstandardized regression coefficient. The bold values provided in table are intended to signal significant relationships.

As for the second hypothesis, which predicts that the frequency of intervention in rural fires to which the firefighters participated will have a moderating effect on the relationship between transformational leadership and the levels of flourishing presented by the firefighters, to the extent that those with more frequency will display a stronger relationship between the independent variable (i.e., transformational leadership) and the dependent variable (i.e., flourishing) than firefighters with less frequency, this hypothesis is partially supported. The moderation effect is only significant (see Table 5), in the interaction between *individualized consideration* and the number of rural fires in which the firefighter was engaged (*β* = 0.02, *p* < 0.05), meaning that only one of the four tested moderation effects become significant. As for the remaining analyzed dimensions, no other significant interaction was found (see Tables 2–4).

**Figure 1:**
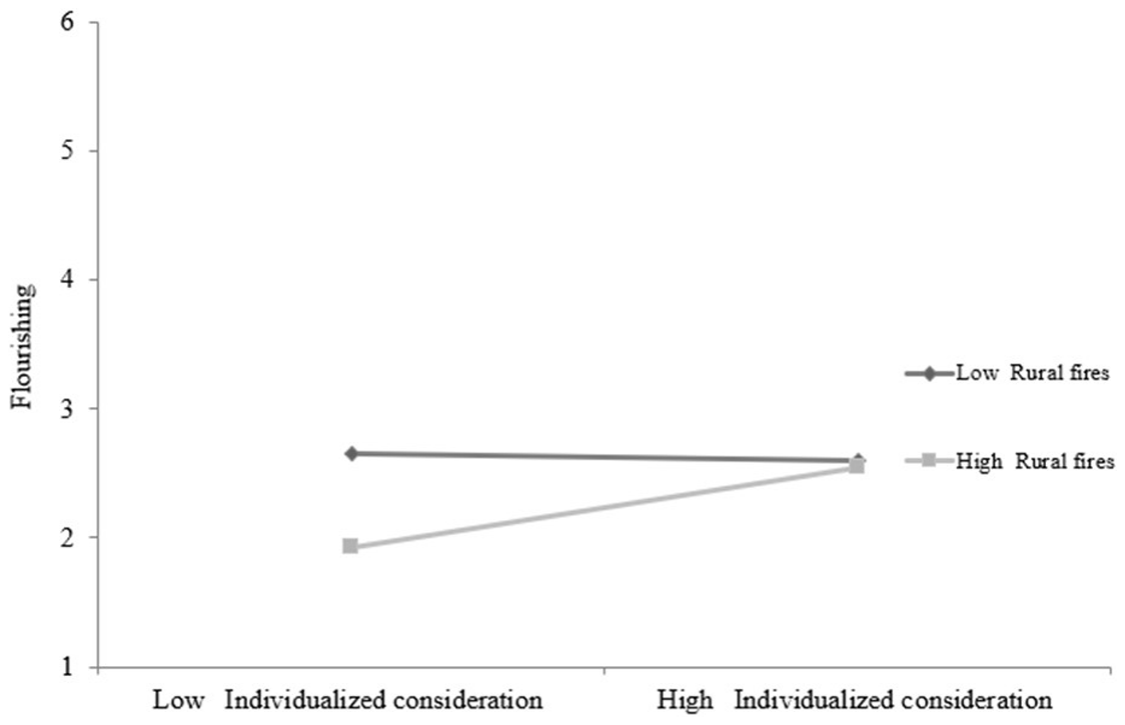
Representative graphic of the interaction between Individualized consideration and Rural fires, and its effect on flourishing.

Figure 1 shows the interaction between the *individualized consideration* dimension of transformational leadership and the moderating rural fires variable and its effect on the flourishing dependent variable. As expected, the graph indicates that in a situation of low intervention in rural fires, the levels of flourishing are identical whether the leader exhibits a high or low level of *individualized consideration*, however when there is high participation in rural fires, the more *individualized consideration* displayed by the leaders the more flourishing is experienced by the subordinates. Thus, *individualized consideration* appears to be a source of resources required by firefighters to face the operational demands related to fighting rural fires, so that flourishing may increase with more *individualized consideration* on the part of the leaders.

## Discussion and conclusion

This study empirically analyzed how the dimensions of transformational leadership affect the perception of flourishing among Portuguese firefighters, as well as the role of the frequency of intervention in rural fires in this relationship. As expected, the results show that: (a) transformational leadership behaviors (i.e., *motivational inspiration*, *intellectual stimulation*, and *individualized consideration* dimensions) positively affect firefighters’ levels of flourishing and (b) in the specific case of the *individualized consideration* dimension, increased operational participation in rural fires strengthens this relationship.

As expected, the transformational leadership revealed a direct and positive (albeit small) relationship with flourishing. Thus, the results presented corroborate the theoretical assumptions in the literature (e.g., Dodge et al., 2012; Arnold, 2017) and similar results have been found in several empirical studies in which transformational leadership is found to be positively related to the promotion of workers’ health and well-being (e.g., Arnold et al., 2007; McMurray et al., 2010; Jacobs et al., 2013). Furthermore, according to Franke and Felfe (2011), transformational leadership dimensions may imply a differential impact on workers’ perceived tension. In fact, in this study, the *idealized influence attributed* dimension is not related with firefighters’ flourishing. This result may be because in this context of emergency there is a culture with high moral and ethical standards assumptions that regulate the decisions and in rural fires firefighters’ do not need the leader as a model to know what behavior to adopt. Thus, the firefighters flourishing levels in a situation with high demands does not dependent on the resources provided by *idealized influence* of leader (Hannah et al., 2009). This hypothesis requires further research with emergency samples (e.g., firefighters, militaries, and polices).

An interesting result was the fact that it was observed that the different dimensions of transformational leadership are not related to the flourishing of the firefighters evaluated at the same moment, but after about 3 weeks. This result seems to reinforce the need to develop longitudinal studies (Arnold, 2017). However, this observation also needs to be analyzed in future research to check if this effect is a methodical artifact.

To some extent, the study also corroborates the importance of the operational demands to which the firefighters are subjected (e.g., the frequency of intervention in rural fires) and their influence on the well-being of these professionals. As expected, rural fires are central demands in the profession under analysis, to the degree that it is in these situations that transformational leadership behaviors are more necessary and valued as well-being promoters. This evidence is in line with that advanced by the literature (e.g., Bakker and Demerouti, 2007; Cowlishaw et al., 2020) and is empirically supported by studies that have analyzed similar variables (e.g., Ângelo and Chambel, 2015; Hentrich et al., 2017). This study shows that the activity of fighting rural fires specifically emphasizes the effects of transformational leadership *individualized consideration* on firefighters’ flourishing. *Individualized consideration* appears to be geared more toward the individual as opposed to the team (e.g., in the item “treats others as people rather than just as a group member”), and in times of greater operational commitment (i.e., more fires) the firefighters appear to require actions focused on themselves and their personal needs in order to achieve the goals of the team. It is in situations of greater attrition that firefighters need their leaders to act objectively as mentors and coaches so that they feel valued and recognized, as exemplified by the items “I am competent and able to do activities that are important to me” or “people respect me.” This is in line with Yammarino et al. (2010), who state that in this type of context, individualized leadership with attention and support actions promotes investment and generates benefits for all parties, namely an increase in subordinates’ self-esteem. Similarly, Baran and Scott (2010) emphasize that in dangerous situations, leadership actions individually targeting firefighters are essential for them to cope with the demands of these extreme events. This evidence reinforces the importance of establishing dyadic relationships between leader and subordinate in these highly demanding professional environments, so that their high levels of flourishing are maintained.

However, no study was found that fully applied this design, where job-related demands acted as a moderator of the relationship between supervisory leadership and a construct related to workers’ well-being. For example, the empirical study of Hentrich et al. (2017) analyzes similar variables but presents job demands as a mediator of the relationship between leadership and workers’ irritation. The line of research followed took into consideration the specific nature of the firefighter profession, where the typology and severity of the situations in which they intervene entail great contextual ambiguity, thus differing from the more stable environments on which most of the research has focused (Baran and Scott, 2010). In the same vein, in their conceptualization of multilevel leadership, based on Bass’ transformational leadership theory, among others, Yammarino et al. (2010) consider crisis or emergency situations such as fighting rural fires as possible moderators of the relationship between leadership variables and well-being indicators.

Contrary to what was expected, the relationship between the remaining transformational leadership dimensions (e.g., *idealized influence attributed*, *motivational inspiration*, and *intellectual stimulation*) and flourishing was not affected by the operational demands under study, i.e., these dimensions do not depend on rural fires frequency to affect the firefighters’ flourishing levels. This result may be due to the fact that the participants in this sample showed high levels of flourishing, which may mean that they already had significant resources to adapt to the additional demands of increased operational demands. This also is in line with Hannah et al. (2009), i.e., when shifting to a more demanding context there may be fewer leadership needs if the resources have been provided in the preparation phase, which may mitigate the effect of increased operational demands on the relationship between the transformational leadership dimensions and the firefighters’ flourishing levels.

### Implications

#### Theoretical implications

These results confirm the COR theory assumption that transformational leadership is a valuable resource to maintain subordinates’ levels of well-being, namely in emergency professionals (i.e., firefighters) who face many demands.

#### Practical implications

This study presents some results that may have implications for this type of highly dangerous context, mainly for the promotion of firefighters’ occupational health. Prior to the period of greater operational commitment, supervisory transformational leadership behaviors are found to have a positive impact on firefighters’ flourishing levels after these operational demands have been met. Thus, it is important that conditions are provided so that supervisors may focus their command action on transformational leadership behaviors, namely in periods prior to greater workloads. To this end, training programs could be developed, as described below, for supervisors to transversally strengthen their competencies in this area: (1) adapted to existing command levels, with the involvement of senior managers; (2) integrating simulations of practical application with group dynamics for knowledge consolidation; (3) providing coaching to prepare participants for change; and (4) planning the application of internal and external validation activities in order to assess whether the content covered is being used in practice (Warrick, 2011). In the selection processes of candidates for leadership positions, the behavioral profiles characteristic of transformational leadership should also be taken into account.

During the most demanding periods, usually the summer months, which coincide with the highest number of rural fires, it is essential that firefighter leaders, who are especially dedicated to this mission, display behaviors geared toward identifying the individual needs of their subordinates, and consequently meeting those needs (Milyavskaya and Koestner, 2011). To this end, the following measures are suggested: (1) proximity in the leader-subordinate relationships, creating an organizational climate that fosters communication and promotes the sharing of concerns, interests and aspirations (e.g., Carr et al., 2003; Zohar and Tenne-Gazit, 2008); (2) provision of feedback to firefighters on the importance and impact of their work for other people (Grant, 2007); and (3) a post-action review session for discussion on what happened and how it can be improved (e.g., Hagemann and Kluge, 2013).

### Limitations and suggestions for future studies

This study presents some limitations. The first is that transformational leadership and flourishing were both assessed by a questionnaire, which favors the common method error. However, a CFA was performed, confronting the theoretical model with the one-factor model. The theoretical model was found to have a significantly better fit to the data than the one-factor model. Therefore, this limitation was safeguarded. Moreover, the data were collected in two waves in this study, also decreasing error-probability. The second limitation is related to social desirability, since the responses concerning transformational leadership were only provided by the subordinate firefighters, which may have influenced the results. It would be interesting in future studies to also measure leadership behavior from the point of view of the supervisors and not only from the perspective of the subordinates (Podsakoff et al., 2003). Thirdly, the small sample size may be a limitation as it may have influenced the number of statistically significant relationships, thus the results obtained should be interpreted with caution. Moreover, the sample is no representative of Portuguese firefighters and does not permit the results’ generalization. Additionally, the fourth limitation is related to the short time span between T1 and T2. Future studies might follow the suggestions of Hannah et al. (2009) who argue that the post event is a critical moment and susceptible to problems with the health of those involved. We suggest that in a future longitudinal study this period should be extended to create more data collection waves, so that it is possible to ascertain how the levels of flourishing evolve following a period of high operational demands (i.e., intervention in many rural fires). It would be equally interesting to analyze in the future how transactional leadership behaviors (e.g., praise and commendations) in this post-event may affect the well-being of the workers (Schriesheim et al., 2006). The fifth limitation is that it was used the frequency of rural fires (an objective measure) as the operationalization of operational demands, thus, not referring to a construct. However, Crawford et al. (2010) distinguished between demands appraise as challenges (that have the potential to promote personal growth or future gains) and appraise as hindrances (that are stressors) that have, respectively, positive and negative effects on workers’ well-being. Therefore, in the future, it would be beneficial to complement objective measures and firefighters’ perception of demands. Moreover, it only was measured the frequency of rural fires and the scope of the operation was not record. Thus, future studies should also include this characteristic that could have influence the firefighters’ demands perception. One last limitation concerns the risk of alpha error accumulation, which is not controlled for in the tested moderation effects. Future studies should take this methodological issue into account.

## Data availability statement

The original contributions presented in the study are included in the article/supplementary material, further inquiries can be directed to the corresponding author.

## Ethics statement

The studies involving human participants were reviewed and approved by the Commission of Ethics and Deontology of Faculty of Psychology, University of Lisbon. The study was conducted according to the guidelines of the Declaration of Helsinki. The patients/participants provided their written informed consent to participate in this study.

## Author contributions

AM was involved in the design, data analysis, and writing and the original draft preparation of this paper. MC was involved in data collection, data analysis, and in the reviewing and editing process. LC was involved in the reviewing and editing process. All authors contributed to the article and approved the submitted version.

## Funding

This work received Portuguese national funding from FCT – Fundação para a Ciência e a Tecnologia, I.P, through the research project with the ref. PCIF/SSO/0054/2018.

## Conflict of interest

The authors declare that the research was conducted in the absence of any commercial or financial relationships that could be construed as a potential conflict of interest.

## Publisher’s note

All claims expressed in this article are solely those of the authors and do not necessarily represent those of their affiliated organizations, or those of the publisher, the editors and the reviewers. Any product that may be evaluated in this article, or claim that may be made by its manufacturer, is not guaranteed or endorsed by the publisher.
